# Projected Wine Grape Cultivar Shifts Due to Climate Change in New Zealand

**DOI:** 10.3389/fpls.2021.618039

**Published:** 2021-04-21

**Authors:** Anne-Gaelle E. Ausseil, Richard M. Law, Amber K. Parker, Edmar I. Teixeira, Abha Sood

**Affiliations:** ^1^Manaaki Whenua — Landcare Research, Wellington, New Zealand; ^2^Manaaki Whenua — Landcare Research, Palmerston North, New Zealand; ^3^Faculty of Agriculture and Life Sciences, Lincoln University, Lincoln, New Zealand; ^4^Plant and Food Research, Lincoln, New Zealand; ^5^National Institute of Water and Atmospheric Research, Wellington, New Zealand

**Keywords:** adaptation, cumulative thermal time, climate change, cultivar, wine grape, phenology

## Abstract

Climate change has already been affecting the regional suitability of grapevines with significant advances in phenology being observed globally in the last few decades. This has significant implications for New Zealand, where the wine industry represents a major share of the horticultural industry revenue. We modeled key crop phenological stages to better understand temporal and spatial shifts in three important regions of New Zealand (Marlborough, Hawke's Bay, Central Otago) for three dominant cultivars (Merlot, Pinot noir, and Sauvignon blanc) and one potential new and later ripening cultivar (Grenache). Simulations show an overall advance in flowering, véraison, and sugar ripeness by mid-century with more pronounced advance by the end of the century. Results show the magnitude of changes depends on the combination of greenhouse gas emission pathway, grape cultivar, and region. By mid-century, in the Marlborough region for instance, the four cultivars would flower 3 to 7 days earlier and reach sugar ripeness 7 to 15 days earlier depending on the greenhouse gas emission pathway. For growers to maintain the same timing of key phenological stages would require shifting planting of cultivars to more Southern parts of the country or implement adaptation strategies. Results also show the compression of time between flowering and véraison for all three dominant cultivars is due to a proportionally greater advance in véraison, particularly for Merlot in the Hawke's Bay and Pinot noir in Central Otago. Cross-regional analysis also raises the likelihood of the different regional cultivars ripening within a smaller window of time, complicating harvesting schedules across the country. However, considering New Zealand primarily accommodates cool climate viticulture cultivars, our results suggest that late ripening cultivars or extended ripening window in cooler regions may be advantageous in the face of climate change. These insights can inform New Zealand winegrowers with climate change adaptation options for their cultivar choices.

## 1. Introduction

Climate change poses major challenges to the wine industry. Research on past observations have shown that in recent decades, climate change has led to advances in phenology, higher sugar concentrations at harvest and compressed or earlier harvests, as well as changes in yield and risk profile (Chuine et al., 2004; Duchêne and Schneider, 2005; Webb and Barlow, 2007; Garćıa de Cortázar-Atauri et al., 2010; Molitor et al., 2014a; van Leeuwen and Darriet, 2016; Wolkovich and Morales-Castilla, 2019). For instance, if a grape cultivar ripens too early, véraison to harvest may coincide with the hottest period of the season which potentially leads to negative effects on flavor, aroma, and alcohol content of grapes (van Leeuwen and Seguin, 2006; Duchêne et al., 2010). One of the most critical climatic drivers accelerating phenology is warmer temperature over the full cycle of development (Jones et al., 2005a,b; Jones, 2012; Cook and Wolkovich, 2016; van Leeuwen and Darriet, 2016; Schultze and Sabbatini, 2019).

This issue is particularly relevant for New Zealand where there is little cultivar diversification and significant regional concentration of production. In 2019, the value of New Zealand wine exports was nearly $1.8 billion (New Zealand Winegrowers, 2020) making wine the second-most significant horticultural export in New Zealand, contributing to 30% of horticultural produce exports in 2019 by value (Ministry for Primary Industries, 2020). Eighty percentage of the total grape production area in New Zealand is a combination of three major cultivars: Sauvignon blanc, Pinot noir, and Merlot (New Zealand Winegrowers, 2020). The growth in area has been considerable in recent years going from 23 to 31 thousand hectares for the period 2010-2019. The Marlborough wine region alone represents 70% of the national production area (New Zealand Winegrowers, 2020), of which 85% of this region's production area is Sauvignon blanc, as well as the region being the largest production area of Pinot noir (Wine Marlborough, 2019).

Phenological modeling can deliver valuable insights into the potential strategies for adaptation to climate change. Research into modeling phenological changes now enables simulations of the time to flowering and véraison (GFV; Grapevine Flowering Véraison model; Parker et al., 2011, 2013) and the time to target sugar ripeness (GSR; Grapevine Sugar Ripeness model; Parker et al., 2020a) for a wide range of cultivars. These models are driven by temperature which allows cultivar suitability to be investigated under the warmer conditions of future climate scenarios across a given country or winegrowing region. The use of models such as GFV and GSR provides a more dynamic way to quantify developmental progression rather than using fixed harvest dates which can be influenced by multiple factors such as end use (e.g., sparkling wine harvest at lower sugar concentrations), logistics of harvest or disease pressure (Garćıa de Cortázar-Atauri et al., 2010). Although Morales-Castilla et al. (2020) characterized increased cultivar diversity for NZ under future climate scenarios, this was not at the scale of individual wine regions. Therefore, using the GFV and GSR models to simulate the timing of key phenological stages at regional scale is of interest to understand cultivar choices as an adaptation strategy to climate change.

In this study, we simulated flowering, véraison and target sugar ripeness (represented as the time to reach a 200 g/l target sugar concentration) using the GFV and GSR models at national scale for a range of cultivars. We considered six global circulation models to project future climate, applied on a 0.05° latitude/longitude grid (approximately 4–5 km at New Zealand latitudes) (Tait et al., 2006). The analysis focused on two time periods for mid-century (2046–2065) and late century (2081–2100). Cultivar suitability and implications for the wine industry were explored by analyzing spatial and temporal shifts in phenology. Results focus on three key regional cultivars of importance to New Zealand's wine industry (Sauvignon blanc, Pinot noir, and Merlot) and one potential future later-ripening cultivar (Grenache) to explore the intra- and cross-regional impacts under different climate change scenarios.

## 2. Materials and Methods

### 2.1. Vineyard Suitable Area

**Figure 1:**
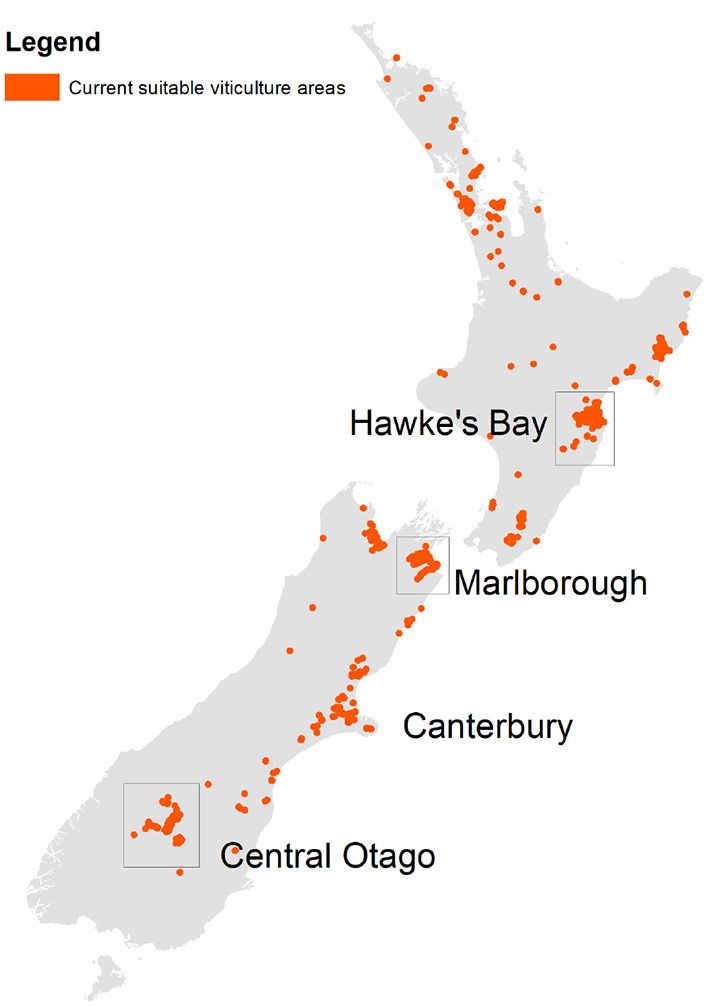
Indicative extent of vineyards in New Zealand (baseline, i.e., 2004). Highlighted areas are three major viticultural regions considered.

Land suitable for viticulture was identified with a combination of a 5 km expansion of the 2004 distribution of viticulture in New Zealand (Manaaki Whenua – Landcare Research, 2004), constrained by a land use capability (LUC) classification (Manaaki Whenua – Landcare Research, 2018) to ensure that land is not affected by fundamental characteristics that would preclude viticulture (e.g., a high water table, or a hazardous area). The dataset was resampled to match the 0.05° climate dataset used to perform the climate change scenario analysis (see Supplementary Material for more details). Figure 1 demonstrates the extent of general suitability for viticulture in New Zealand given this definition.

Three key wine producing regions were considered: Hawke's Bay, Marlborough, and Central Otago (Figure 1). Marlborough is the largest producing region, with Sauvignon blanc representing 76% of national production (New Zealand Winegrowers, 2020). Central Otago is the southernmost growing region in NZ and has an important production area of Pinot noir (80% of Central Otago viticultural areas), the second most planted grape cultivar in NZ. Hawke's Bay represents the second biggest production area in NZ of which Merlot is an important red cultivar (New Zealand Winegrowers, 2020). Internationally the three cultivars selected in this study are within the top 10 planted cultivars worldwide (Anderson and Aryal, 2013).

### 2.2. Climate Change Projections

Climate outcomes are based on a set of Representative Concentration Pathways (RCP) representing scenarios for approximate total radiative forcing at 2100, relative to 1750. The fifth IPCC assessment (Intergovernmental Panel on Climate Change, 2014) selected four scenarios referenced as RCP 2.6, 4.5, 6.0, and 8.5 (for respective radiative forcing of 2.6, 4.5, 6.0, and 8.5 W.m^-2^). Six Global Circulation Models (GCM) from the Coupled Model Inter-comparison Project (CMIP5), were previously used to downscale simulations of the RCP for New Zealand (Tait et al., 2016; Ministry for the Environment, 2018). The GCMs considered in the analysis were BCC-CSM1.1, CESM1-CAM5, GFDL-CM3, GISSE2-R, HadGEM2-ES, and NorESM1-M. The output variables from these models included daily precipitation, maximum and minimum daily air temperature, daily average relative humidity, daily average solar radiation, and daily average wind speed at 10 m. Daily mean temperatures for the period 1979–2120, considering three mean temperatures were calculated as the arithmetic mean of the minimum and maximum daily temperatures from datasets downscaled by the New Zealand National Institute of Water and Atmospheric Research (NIWA).

The three periods of interest were determined as current (1971–2005), mid-century (2046–2065), and end of century (2081–2100).

A summary of current and future temperature change in the three regions of interest is shown in Table 1. The range of shifts in temperature is around 0.5 to 3 °C warmer depending on the RCP. Optimal temperature for grapes are around 30 °C, with signs of heat stress above 35 °C (Kliewer, 1977; Hunter and Bonnardot, 2011; Hochberg et al., 2015). For the projections considered in this study, this threshold of maximum temperature was only crossed up to 1.9 days/year under RCP 8.5 by the end of the century in the Otago region.

**Table 1:** Summary of current temperature (from https://niwa.co.nz/our-science/climate/publications/regional-climatologies) and projections range (for RCP 2.6–8.5) in the study regions (Ministry for the Environment, 2018).

**Region**	**Current average daily temperature (**^****°****^**C)**		**Mid-century (compared to 1995)**	**End of century (compared to 1995)**	**Mean regional number of days where maximum temperature is over 32^**°**^C (end of century, RCP 8.5)**	**Mean regional number of days where maximum temperature is over 35^**°**^C (end of century, RCP 8.5)**


	**Winter**	**Summer**				
Marlborough	2–3	21–23	+0.7–1°C	+0.7–3°C	2.4 days/year	0.27 days/year
Hawke's Bay	3–5	21–24	+0.7–1.1°C	+0.7–3.1°C	5.6 days/year	0.4 days/year
Otago	-4–+1	19–22	+0.6–0.9°C	+0.6–2.8°C	17.8 days/year	1.87 days/year

### 2.3. Phenology Projection

We used the GFV model developed by Parker et al. (2011) to simulate the time of key phenological stages of flowering and véraison defined as the time at which 50% capfall had occurred and when 50% of berries softened or changed from green to translucent for white cultivars or changed color from green to red for red cultivars (Parker et al., 2011). This model is a linear growing degrees days model that relates cumulative daily temperature to the day of the year (DOY) when flowering or véraison occurs. The thermal summation (F*) is specific to each cultivar. In the case of the GFV model, the cumulative summation starts on the 60th DOY in the Northern Hemisphere (*t*_0_) corresponding to the 242nd DOY in the Southern Hemisphere and uses a base temperature (*Tb*) of 0 °C. The Grapevine Sugar Ripeness (GSR) model (Parker et al., 2020a) is also a linear model used to simulate the time to reach a target sugar concentration in the berries. The GSR model has *Tb* = 0 and *t*_0_ = 273 (Southern Hemisphere). A target sugar concentration of 200 g/l was selected because it represents a mid-target sugar concentration provided within the range presented in Parker et al. (2020a), and it is close to that of an accepted target for Sauvignon blanc (21.5°Brix) as determined by winegrowers in Trought and Bramley (2011).

The corresponding DOY values of flowering, véraison (using GFV) and target sugar ripeness (using GSR) per year were calculated for selected cultivars and F* values, derived from (Parker et al., 2013, 2020a) (Table 2). These raster grids were recorded in a static database for all RCPs and GCMs, with an output resolution of 243x260 pixels (same as the input climate data), covering all of mainland NZ. To summarize the change in phenology for future periods, we recorded the simulated flowering, véraison and target sugar ripeness for 2046–2065 to represent mid-century, and 2081–2100 to represent end of century. For each region of interest, we then summarized the median DOY across the region, for each GCM and each year within the three periods (current, mid-century, and end of century). Areas not capable or not suitable for wine production were masked out for all periods.

**Table 2:** F* values for four cultivars, Pinot noir, Merlot, Sauvignon blanc, and Grenache as determined by the GFV model (Parker et al., 2011, 2013) for flowering and véraison, and GSR model (Parker et al., 2020a) for the time to 200 g/l sugar concentation.

	**Flowering**	**Véraison**	**Sugar concentration 200 g/l**
Pinot noir	1 219	2 511	2 838
Merlot	1 269	2 636	2 856
Grenache	1 277	2 761	2 967
Sauvignon blanc	1 282	2 528	2 820

The GFV and GSR were selected for the study because they represent some of the most extensively calibrated and validated phenological models to date for the grapevine (Parker et al., 2011, 2020a). The GFV database (which was divided into calibration and validation data) consisted of observations from 1960 to 2007, from 123 locations including 12 of the principal viticulture regions of France, Changins in Switzerland, Veneto and Tuscany in Italy, and the Peloponnese region in Greece, and 81 cultivars. This equated to 2 278 flowering observations and 2 088 véraison observations (Parker et al., 2011). The GSR database covered six different target sugar concentrations, spanning the period 1963–2014, nine of the principal viticulture regions of France, as well as Germany, Greece, Italy, Luxembourg, Portugal, Spain, and Switzerland, and 65 cultivars. Depending on the target sugar concentration there was up to 1 223 observations (quantity used for calibration and validation for 170 g/l sugar concentration) (Parker et al., 2020a). To date, these models represent the most spatially and temporally robust temperature-based models currently in application for phenological predictions that can be used in new sites, new climates, or new cultivars for the grapevine. The GFV model and the GSR model have also been validated in the New Zealand context for Marlborough Sauvignon blanc, the cultivar for which there is extensive data available (Parker et al., 2014, 2015, 2020b). In the most recent validation (Parker et al., 2020b), the model was tested for the period 2004–2020 and it was found the goodness-of-fit (root mean squared error) was 6.67, 4.67, and 9.67 days for flowering, véraison and time to 200 g/l sugar concentrations respectively which was within prediction ranges of the original calibrations and validations of cultivars by the models (Parker et al., 2011, 2013, 2020a). In addition, the temperature relationships for the GSR and GFV models have been successfully validated beyond the original model development datasets (Verdugo-Vásquez et al., 2019; Parker et al., 2020b; Ramos and de Toda, 2020; Wang et al., 2020) including other cool climate areas such as Champagne (Parker et al., 2020a) and areas in China (Wang et al., 2020). They have also been applied in combination with future climate change projections (van Leeuwen et al., 2019; de Rességuier et al., 2020; Parker et al., 2020b; Ramos and de Toda, 2020). Together, these tests and applications of the GSR and GFV models illustrate the models' broad applicability to new sites and different climates.

For cross-regional analysis, the combined overall median range for the time of flowering, véraison, and target sugar ripeness for the current period was determined for Sauvignon blanc (Marlborough), Hawke's Bay (Merlot), and Pinot noir (Otago) as well as the combined overall projected median range for all RCPs. The cultivar-by-region projections were then compared among the regions and the different RCPs.

## 3. Results

### 3.1. Current and Projected Phenology at Regional Scale

Few differences among GCMs were observed for any given RCPs for flowering, véraison, and time to 200 g/l sugar concentration of the three cultivar-region combinations of interest (Supplementary Material). As the GCM variability tended to be similar across temporal scale (mid- to end of the century), we used the mean GCM to summarize our findings. Since conclusions can be drawn in the three regions of interest, we presented results for Marlborough, with results for Hawke's Bay and Central Otago available in Supplementary Material.

**Figure 2:**
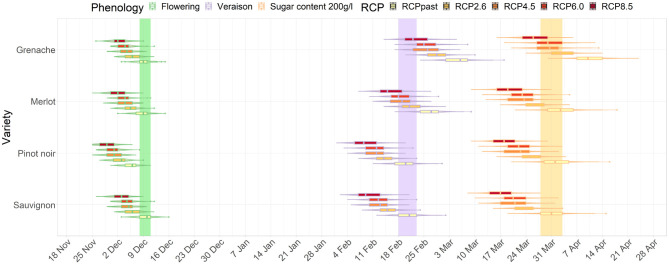
Distribution of dates for flowering, véraison, and sugar levels reaching 200 g/l for Sauvignon blanc, Pinot noir, Merlot and Grenache in Marlborough for the mid-century period. The colored bars show the current range of dates for Sauvignon blanc.

**Figure 3:**
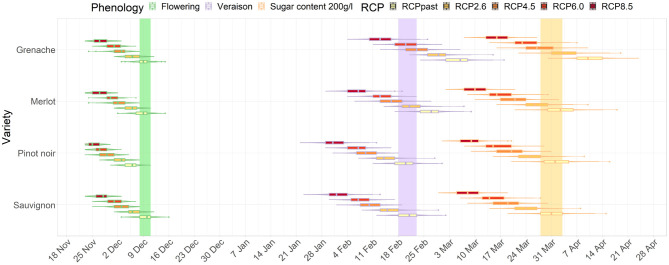
Distribution of dates for flowering, véraison, and sugar levels reaching 200 g/l for Sauvignon blanc, Pinot noir, Merlot and Grenache in Marlborough for the end-of-century period. The colored bars show the current range of dates for Sauvignon blanc.

For the average GCM data, the magnitude of difference between future and current dates in timing of phenological stages progressively increased with increased radiative forcing (Figures 2, 3). The differences between RCPs also increased for later phenological stages. For example, the shift in flowering was similar across RCP scenarios for flowering for Sauvignon blanc (4 to 7 days difference from current period) by the mid-century. However, differences between RCPs were larger for véraison and for target sugar ripeness occurring 14 and 16 days earlier for RCP 8.5 compared with RCP 2.6. Results clearly show a compression of time between flowering and target sugar ripeness corresponding with higher greenhouse gas emissions pathways.

In Marlborough, flowering dates for all cultivars and RCPs advanced earlier than the current flowering window for Sauvignon blanc (green bar on Figure 2). Pinot noir, which is also extensively planted in the Marlborough region, in a high emission (RCP 8.5) scenario, flowering advanced 7 days. The timing of véraison advanced for all RCPs for Pinot Noir and Sauvignon blanc in a similar range (purple bar on Figure 2). Merlot, which usually has a later véraison date than Sauvignon blanc, reached a similar véraison period as a current Sauvignon blanc under RCP 2.6 and 4.5. However, for Grenache, the timing of véraison overlapped the current Sauvignon blanc véraison period only in the case of RCP 8.5 (Figure 2).

For the mid-century projections target sugar ripeness, Pinot noir and Merlot showed similar magnitudes of advancements to Sauvignon blanc. All RCPs' projections for Grenache overlapped with the current period defined for Sauvignon blanc (orange bar on Figure 2), except Grenache dates were earlier than current Sauvignon blanc dates under RCP 8.5 (Figure 2).

By the end of the century, the projected differences between RCP 2.6 and 8.5 became more dramatic than for the mid-century projections of all three stages of development. Under RCP 2.6, Grenache cultivar reached a target sugar ripeness at a similar time to the current period for Sauvignon blanc. However, under RCP 8.5, all cultivars including Grenache have projected véraison dates and sugar ripeness dates earlier than the current period for Sauvignon blanc. Grenache projections indicated that its target sugar ripeness would be attained 2 to 3 weeks earlier the current period for Sauvignon blanc (Figure 3).

### 3.2. Current and Projected Flowering Dates at Regional Scale

**Figure 4:**
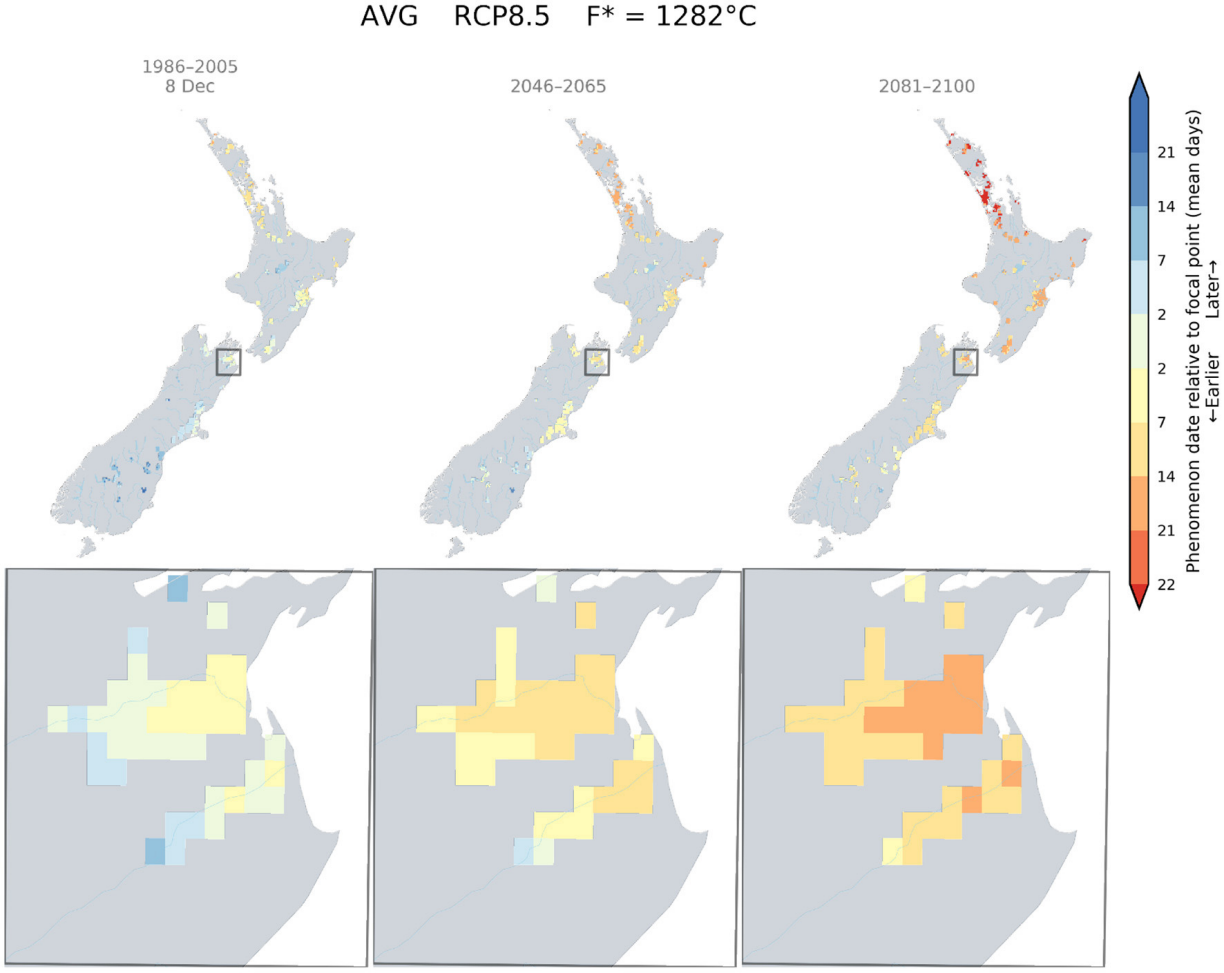
Predicted changes in flowering dates for Sauvignon blanc using the Grapevine Flowering Véraison (GFV) model and scenario RCP 8.5, as an average over all GCMs. The rectangle indicates the Marlborough region, where the principal cultivar is Sauvignon blanc. The focal point was determined as the average flowering date for Sauvignon blanc in the Marlborough region for the current period.

The climate change impacts on phenology for different wine grape cultivars in New Zealand demonstrated advances in dates for flowering, véraison, and the target sugar ripeness for the three key regions: Marlborough, Hawke's Bay, and Central Otago. For a given cultivar and RCP scenario, this shift occurs homogeneously across the studied regions. However, each developmental stage would be reached at a similar time in more southern parts of New Zealand. We examined the date at which flowering occurred currently in Marlborough for Sauvignon blanc (approximately 8th December), and mapped the relative difference between this date and the projected flowering dates that could occur in suitable wine areas of New Zealand for RCP 8.5, using the GFV model. The results indicated that a Sauvignon blanc would reach the 8^th^ of December flowering date in Canterbury and Central Otago by the middle of the century and South Canterbury by the end of the century (Figure 4).

**Figure 5:**
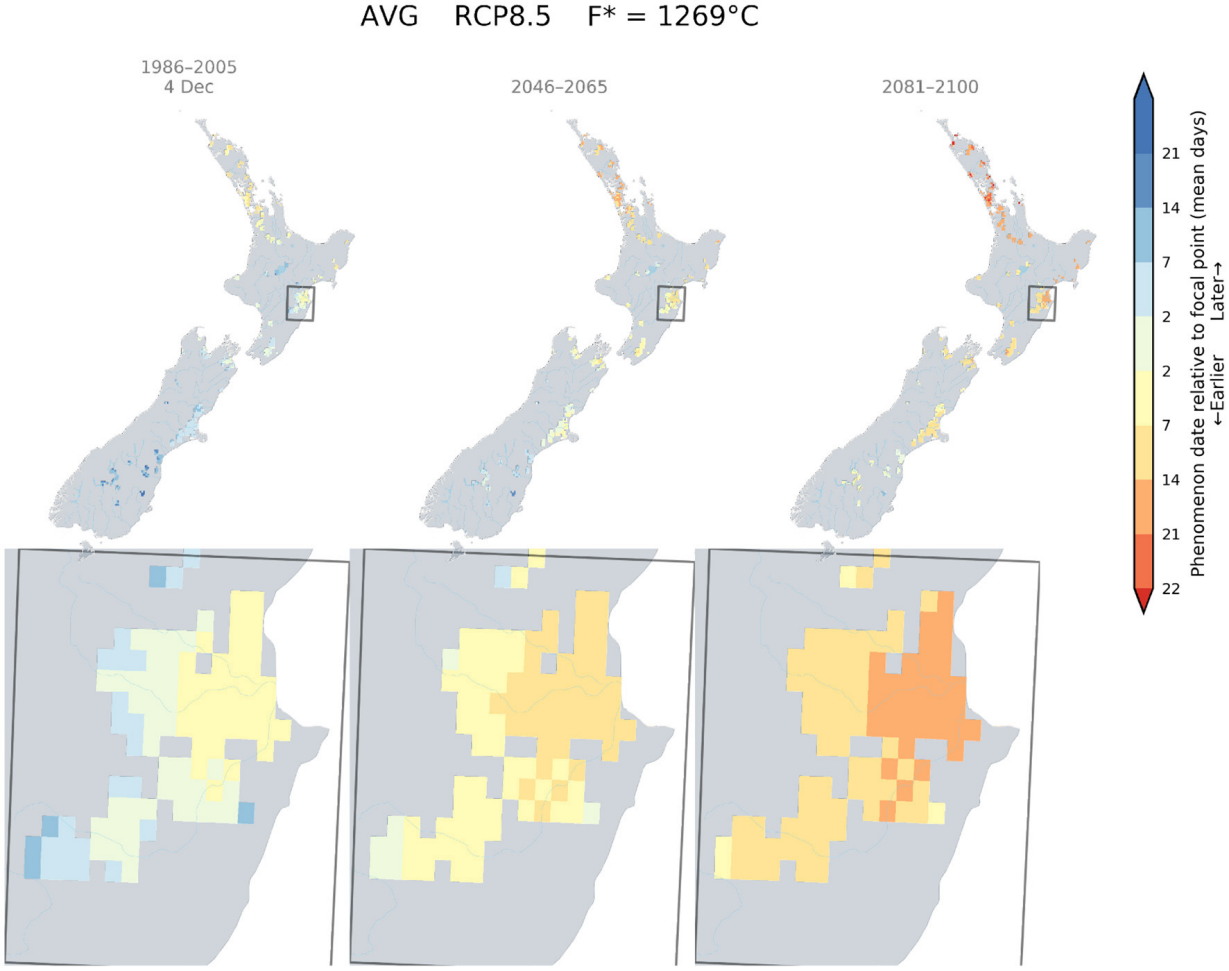
Predicted changes in flowering dates for Merlot using the Grapevine Flowering Véraison (GFV) model and scenarios RCP 8.5, as an average over all GCMs. The rectangle indicates the Hawke's Bay region, where Merlot is mostly grown in New Zealand. The focal point was determined as the average flowering date for Merlot in the Hawke's Bay region for the current period.

Similarly, the current flowering date of Merlot for Hawke's Bay was determined to be around the 4th of December. Because this date is close to the flowering date of a Sauvignon blanc in Marlborough, we also found that a Merlot would flower around the 4th of December in Canterbury by mid-century, and in Central Otago and South Canterbury by the end of the century (Figure 5).

**Figure 6:**
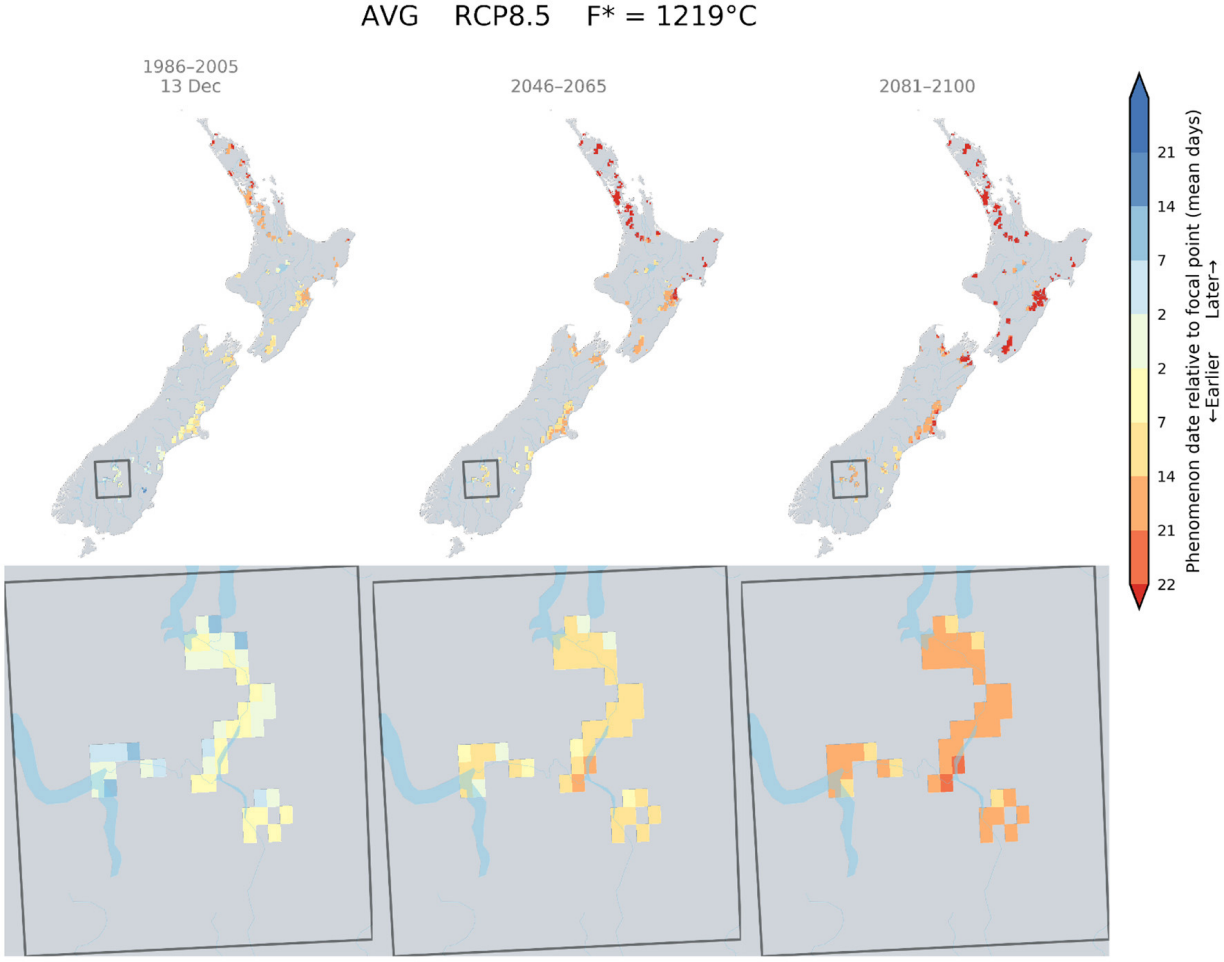
Predicted changes in flowering dates for Pinot noir using the Grapevine Flowering Veraison (GFV) model and scenario RCP 8.5, as an average over all GCMs. The rectangle indicates the Otago region, where the principal cultivar is Pinot noir. The focal point was determined as the average flowering date for Pinot noir in the Otago region for the current period.

Currently Pinot noir in Central Otago was found to flower around the 16th of December. This flowering date was only attainable in South Canterbury by mid-century and a small area of this region by the end of the century (Figure 6).

Similar shifts and spatial patterns were observed for véraison and target sugar ripeness dates (Supplementary Material).

### 3.3. Cross-Regional Analysis

**Figure 7:**
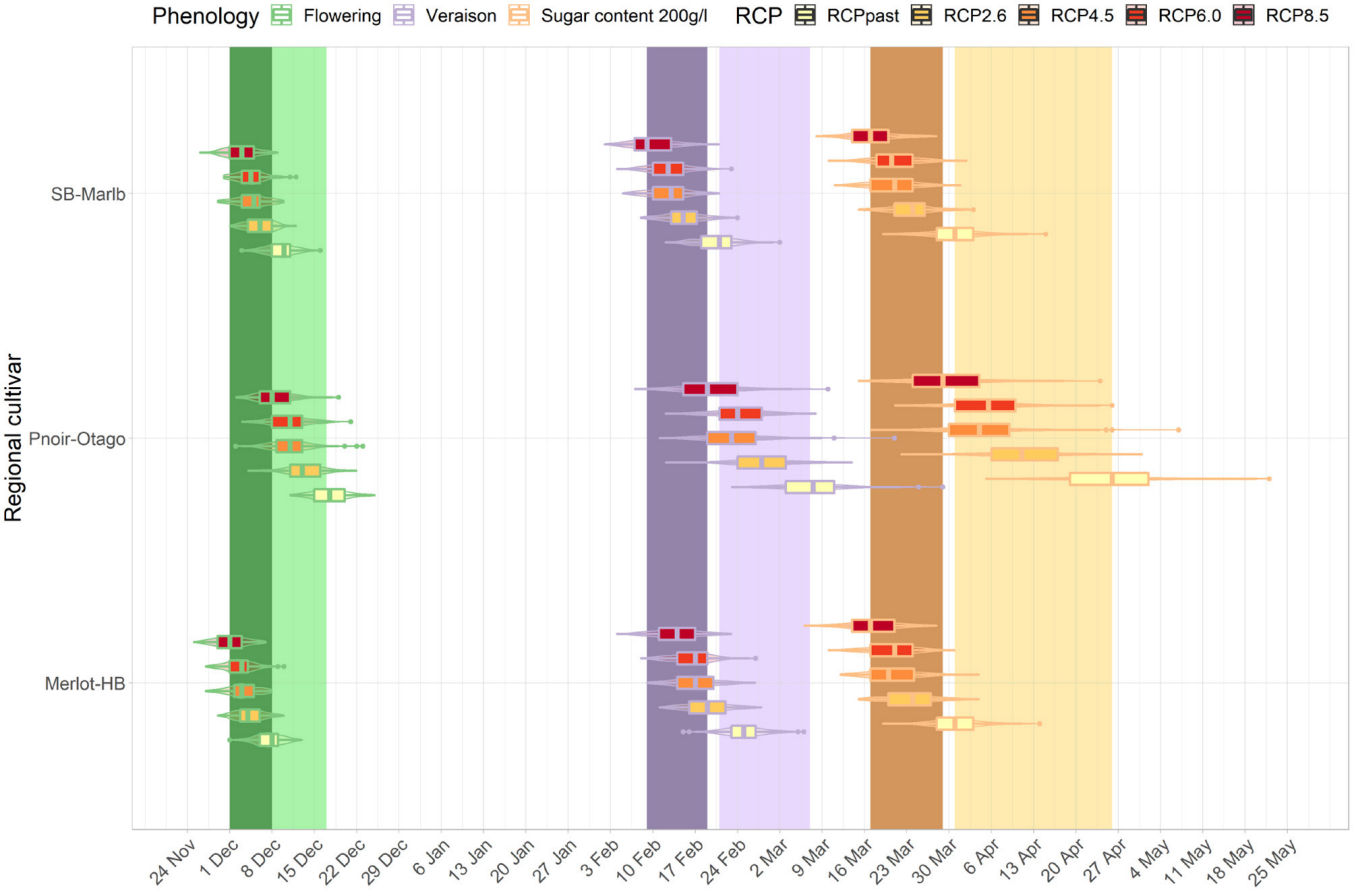
Predicted changes in flowering, véraison, sugar concentration dates for the major cultivars in their associated regions. The box plots correspond to projections for the key cultivar or interest for each region: Marlborough (Sauvignon blanc), Central Otago (Pinot noir), Hawke's Bay (Merlot) for the mid-century period and RCP 2.6, 4.5, 6.0, and 8.5. The colored bars represent the combined range of median dates for the three region-cultivar combinations at each phenological stage associated with flowering (green), véraison (purple) and sugar concentration (orange) for current period (light bars) and RCP 8.5 (dark bars). Combined range of median dates was defined as all median dates obtained for each region and cultivar combination for each year of the current period.

**Figure 8:**
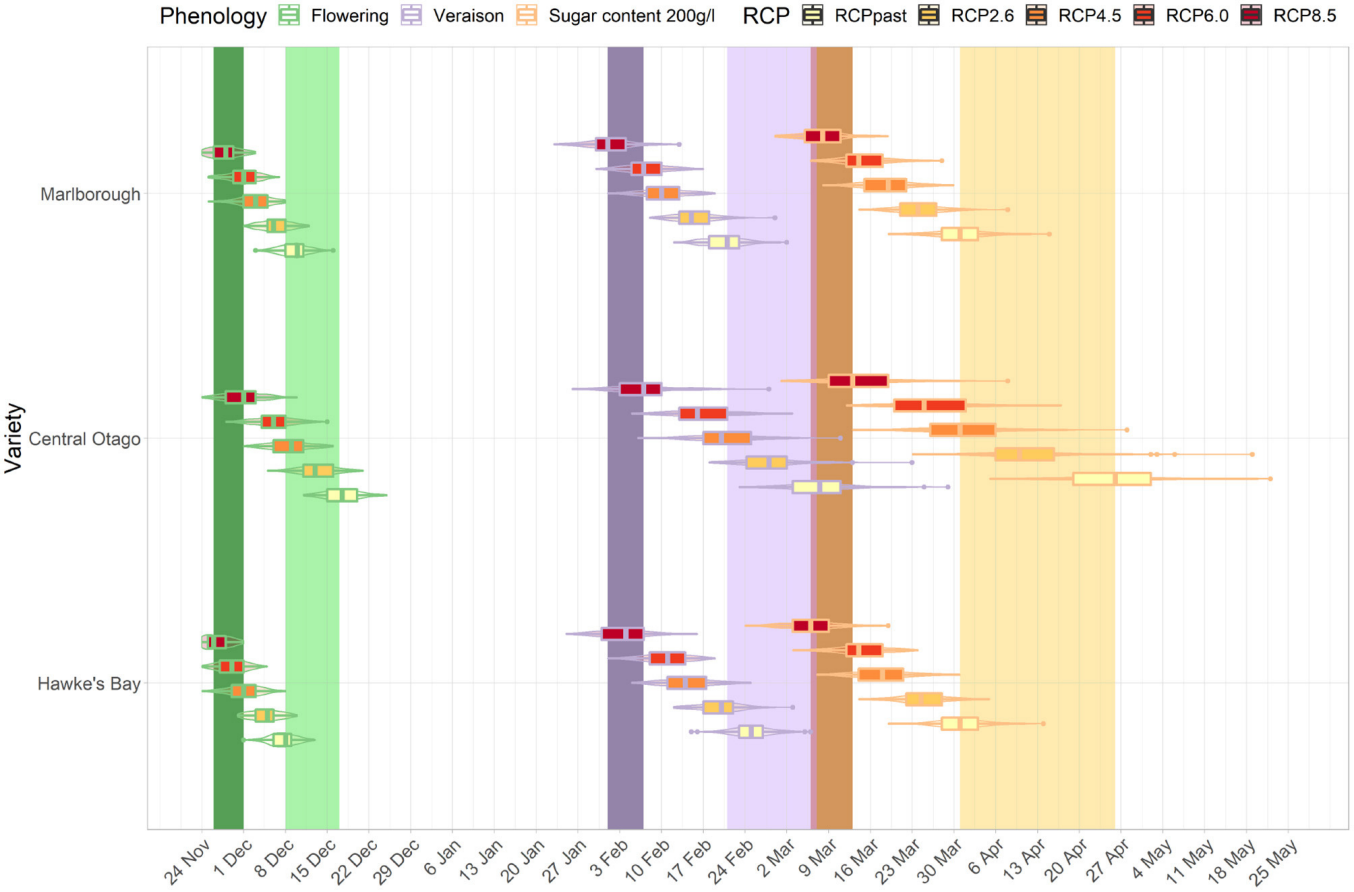
Predicted changes in flowering, véraison, sugar concentration dates for the major cultivars in their associated regions. The box plots correspond to projections for the key cultivar or interest for each region: Marlborough (Sauvignon blanc), Central Otago (Pinot noir), Hawke's Bay (Merlot) for the end-of-century period and RCP 2.6, 4.5, 6.0, and 8.5. The colored bars represent the combined range of median dates for the three region-cultivar combinations at each phenological stage associated with flowering (green), véraison (purple) and sugar concentration (orange) for current period (light bars) and RCP 8.5 (dark bars). Combined range of median dates was defined as all median dates obtained for each region and cultivar combination for each year of the current period.

The development stages for three cultivar-region combinations occur across a wide range of dates in the current period (light bands, Figures 7, 8). For example, the median véraison date occurs around the 17th of February, 24th of February, and 8th of March for a Sauvignon blanc cultivar in Marlborough, a Merlot in Hawke's Bay, and a Pinot noir in Central Otago respectively, creating a window of véraison dates of 19 days. That range of dates compressed for future periods (mid- and end of century, dark bands Figures 7, 8) under RCP 8.5, as véraison would happen for all three cultivars across a shorter period (between the 10th and the 18th of February). The range of dates for target sugar ripeness across the three cultivar-regional combinations compressed from 26 to 12 days by mid-century under RCP 8.5 (Figure 7), down to a 7 day period by the end of the century (Figure 8).

For all cultivar-region combinations, the differences among the RCPs in the projected time of target sugar ripeness were greater than differences among RCP projections for flowering and véraison. When comparing between regions, the advances in timing of the three stages irrespective of RCP were similar for Hawke's Bay Merlot and Sauvignon blanc Marlborough, but Central Otago Pinot was comparatively less advanced and showed greater variation in projections. By the end of the century, the projected time of target sugar ripeness for Merlot in Hawke's Bay and Sauvignon blanc in Marlborough overlapped (boxplot in Figure 8) with the time range of véraison for the three cultivars under RCP 2.6 and 4.5. There was also an overlap for the future timing of target sugar ripeness in Central Otago under RCP 8.5 with its current timing of véraison (light purple band, Figure 8).

## 4. Discussion

The application of the GFV and GSR empirical models on selected New Zealand wine grape cultivars for a range of different RCPs and GCMs allowed us to explore the impacts of climate change on timing of three important crop phenological stages. Our analysis showed that dates for flowering, véraison and target sugar ripeness advanced as a function of warming in the RCP scenarios considered. The differences between high- and low-emissions pathways were more prominent by the end of century. We found greater differences between RCPs for véraison and target sugar ripeness dates compared to flowering dates. This shows that tracking toward a lower RCP would likely minimize the impact on these two key phenological stages. Results also suggest that cultivar shift as a potential adaptation strategy is possible if winegrowers aim to maintain the same window of time for each development stage in the current regions of study. For instance, depending on the emissions pathway, it was shown that a later flowering and ripening cultivar such as Grenache may flower, go through véraison or reach a 200 g/l target sugar ripeness at a similar time to that of currently planted Sauvignon blanc. Similarly, if growers aim to maintain the main cultivars currently grown in New Zealand (Sauvignon blanc, Pinot noir, and Merlot) with similar calendar of phenological stages, a spatial shift of producing areas to more Southern regions would be required in the future, or application of field management to maintain the timing of phenology.

We looked at whether the phenological shifts were occurring at the same rate across the various climate change scenarios for the three main cultivars of interest in each respective region of their growth (Sauvignon blanc in Marlborough, Merlot in Hawke's Bay, and Pinot noir in Central Otago). We showed the rate of change in phenological stages is different between cultivars, a similar conclusion to observations made in Australia (Petrie and Sadras, 2008), resulting in a compression in the range of maturity dates, particularly at higher emission scenarios. For instance, RCP 2.6 shows an even shift in dates for the three cultivars however under RCP 4.5 to 8.5, the timing for reaching phenological stages becomes uneven across the three cultivars. Pinot noir in particular shows a much faster rate of change, resulting in all three cultivars reaching the same stage within up to half its current range of dates by the end of the century. This compression in time may be a concern for the wine industry, as grapes will likely mature at a similar time, thus putting pressure on scheduling of harvesting and transport of harvested grape to facilities.

From a varietal change perspective, a cultivar with later ripening than the currently used ones may become more suitable if the target is to keep the same calendar of phenological events. The workflow developed with the GFV and GSR models can be further extended to explore such adaptive strategies as all the required datasets are now pre-computed, which enables the testing of potential phenological changes across grape cultivars and New Zealand regions for the four RCP scenarios. We also showed that it was possible to use the GFV model to test and select substitutable cultivars, i.e., to target flowering in a region at a specific date to keep existing phenological calendars similar as in historical climate. This could allow, for example, to explore benefits of minimizing drastic shifts in temporal patterns of phenological events.

Our results on spatial phenology stage shifts focused on areas that are currently suitable for viticulture in New Zealand. However, new areas could become suitable under climate change, opening new opportunities for key cultivars in regions of New Zealand that are not traditionally known as wine-growing regions. Under climate change, southern parts of New Zealand that are presently too cool (i.e., a flowering date beyond mid-December) could eventually exhibit earlier flowering dates. Concurrently in these regions, the risks to frost may also become less of a constraint under warmer temperature, although this would need to be combined with earlier timing for budbreak (Mosedale et al., 2015; Sgubin et al., 2018). For a comprehensive assessment of potential gains of production across New Zealand viticulture regions in the future (Morales-Castilla et al., 2020), other aspects influenced by edapho-climatic factors would need to be considered, including water supply, risk of biotic stresses and cultivar responses to photoperiod (Parker et al., 2013).

### 4.1. Scope, Implications, and Limitations of This Study

Although the land use capability (LUC; Manaaki Whenua – Landcare Research, 2018) delineated potential growing regions for this study, it is acknowledged that there may be opportunities to consider expansion to new production areas in the future. Our projections are available across the country at 0.05° resolution, so it is possible to change the extent of potential areas for viticulture. Another important consideration is that the projections are based on temperature only. While temperature is the key driver of phenology (Cook and Wolkovich, 2016), other climatic drivers such as projected rainfall changes may also impact on suitable regions in the future. To further establish the impact of rainfall for determining suitable regions, broader assessment of suitability thresholds for viticulture would need to be considered in future research.

The spatial resolution (0.05° grid) used to perform the climate change scenario analysis enabled us to produce information at the regional level, but it does not account for finer aspects of topography and complex climate interactions that can occur at a lower resolution, particularly in the context of the NZ maritime temperate climate (Parker et al., 2015; Sturman et al., 2017). Given the complex terrain of NZ, the extension of our analysis to finer spatial resolutions would likely provide additional insights on phenological responses. Such research has been carried out in St. Emilion, Bordeaux, and the results indicate that within region variability also plays an important role in future projections (Le Roux et al., 2016; de Rességuier et al., 2020). Future research will benefit from the availability of downscaled input datasets at finer resolutions and analytical methods to aggregate simulations across scales, an active area of research in the field (Ewert et al., 2015).

It is important that model complexity and fit within temperature boundaries are considered when selecting models to address specific research questions in different environments, as more complex model structures with additional parameters could be considered for future improvement. Over-optimal temperatures have a negative impact on plant development, causing a decreased rate of development. Non-linearity and threshold responses of plant development to temperature have therefore been calibrated in other grapevine phenophase models (Garćıa de Cortázar-Atauri et al., 2010; Cuccia et al., 2014; Molitor et al., 2014b, 2020; Morales-Castilla et al., 2020; Prats-Llinàs et al., 2020) and these could be incorporated in the future to enhance our methodological approach. So far, test comparisons between the Wang and Engel (1998) model, which has a temperature threshold, and the GFV model have shown few differences for Pinot noir (Burgundy) (Cuccia et al., 2014) in cool-climate regions that are comparable to New Zealand at degrees of warming of up to 5 °C. Only when considering the warmer region of Seville were minor differences detected between the two models (Cuccia et al., 2014). Besides, curvilinear temperature relationships were tested for both the GSR and GFV models, but the model accuracy did not improve within the mean minimum and maximum temperature of calibration datasets (ranging from 15–32 °C for the GFV, 14–34 °C for the GSR) (Parker et al., 2011, 2020a). As the temperature ranges for the future climate scenarios did not exceed those of the temperatures used for the GFV and GSR model calibration (Table 1), these models were considered fit to assess the different cultivars responses to future temperature in our study. However, the structure and parameterization of phenological models suitable for future climate change studies warrants continual review for any crop, grapevine included, as more information on patterns of response to extreme temperatures is made available to be integrated in models (e.g., Rosenzweig et al., 2013).

The GFV is an empirical model that has been calibrated on phenology date and underlying understanding that temperature is the main driver of change. By projecting the changes in temperature and inferring phenology shifts, some key uncertainties need to be noted. First, the climate change projections showed more variability between GCMs by the end of the century, leading to more uncertainties on the range of shifts in dates. Second, the model itself assumes that the empirical relationship between temperature and phenological development will stay consistent in time. While temperature-based phenological models are used for many plant species for climate change studies, we do not know to what extent other variables might influence future projections. For instance, some studies suggest that CO_2_ concentrations might change the optimum temperature for photosynthesis, and influence biomass production, sugar concentrations, acidity levels, and water use efficiency (Bindi et al., 2001; Schultz and Stoll, 2010). Determining regional suitability for particular cultivars still requires careful consideration of additional variables. For instance, while the period from dormancy to flowering is mainly determined by temperature (and thus adequately captured by the GFV model), the period from flowering to véraison can also be influenced by water deficit (Mart́ınez-Lüscher et al., 2016). Management aspects such as leaf area to fruit weight ratio manipulations (Parker et al., 2014) and the interaction of factors (CO_2_, water stress, and temperature) also need to be considered to fully represent vine physiological responses in the context of climate change (Mart́ınez-Lüscher et al., 2016). Furthermore, climate change may have downstream effects on the suitability of pests and pathogens, changing defence mechanisms of the plant but also the life cycle of some insects (Santos et al., 2020). The increased asynchrony between plant and pest phenology may have both positive and negative impacts (Reineke and Thiéry, 2016). Third, it is valuable to test the variability of these relationships across different soil and climate environments as new datasets are made available. For instance, while our analysis is appropriate to draw conclusions at the regional scale, the influence of soil and micro-climate may require further analysis and downscaled information to understand impacts at a local scale.

The projected shifts in wine grape phenology in New Zealand may have some important implications for biophysical dimensions of production systems. For instance, warmer temperatures as noted in the 2017–2018 heatwave in NZ, resulted in earlier and compressed flowering, improved fruit set and improved bunch initiation, both of which lead to increased yields for the 2017–18 and 2018–19 season (Salinger et al., 2019, 2020). Therefore, in cool climate viticulture production there could be potential production benefits if temperatures do not exceed optimum physiological thresholds. Excessive heat may have adverse consequences for production especially from flowering to véraison stages when berry grapes are most sensitive (Belliveau et al., 2006). The ripening period might shift toward the hotter part of the season, leading to changes in temperatures during the ripening period (Trought et al., 2015), which could not only change grape sugar concentrations, but also flavor and aroma profiles. However, recent research has indicated that in current seasons where phenology has advanced due to heatwaves, a reciprocal increase in temperature during the ripening period did not occur (Salinger et al., 2020). Therefore, understanding the biophysical changes at specific times of the growing season at local and national levels will be important for assessing the implications of climate change on grape and wine production. The projected changes could also have equally important implication for the human dimension of the production system. If temperatures increase, it is not only the impact on grapevines that need to be considered but also the increasing risk to workers' exposure to summer heat at labor-intensive stages (Ioannou et al., 2017). Our projections also show that phenology shifts are uneven across the country, leading to different degrees of compression in time of reaching target sugar concentrations for the three regions of interest. This has implications in preparedness for upgrading infrastructure and creates a shorter harvesting time span causing competition for seasonal laborers (Petrie and Sadras, 2008; Cradock-Henry and Fountain, 2019). Therefore, it is essential to consider grape and wine production in terms of linked social-ecological or human-environmental systems, addressing changes in biophysical processes as well as human and management activities within a complex adaptive system (Berkes and Folke, 1994; Cradock-Henry and Fountain, 2019).

In response to shifts in phenology, winegrowers have several adaptation measures that could be considered. Adaptation strategies are often distinguished short-term, seasonal or interannual responses or tactical adaptation; and longer-term, more strategic actions. In the shorter term, tactical adaptation could be adopted by shifting viticultural techniques to delay ripeness (van Leeuwen et al., 2019). Often these techniques such as leaf area manipulations or delayed pruning only delay ripeness by 1–3 weeks (Friend and Trought, 2007; Parker et al., 2014; Petrie et al., 2017; Moran et al., 2019). Depending on the RCP scenario considered, this may be insufficient for successful adaptation and more strategic, even transformational changes may needed (Fleming et al., 2015). In the longer term, a key adaptation strategy to climate change for wine growers remains to change cultivars to buffer winegrowing regions' losses under future climate conditions (Morales-Castilla et al., 2020). Flexibility in cultivar choice allows winegrowers to maintain harvest dates close to optimal windows and to enhance resilience to climate change impacts (van Leeuwen et al., 2019). By managing harvest timing, growers can influence environmental conditions prevalent during ripening. The choice on new cultivars may also be reviewed in combination with other desired adaptation management decisions (such as rootstock height, trunk height or leaf area to fruit ratio or delayed pruning) that would increase resilience to other climate-change related stressors such as droughts, storms, or heat stress. Increasing genetic diversity in a crop may also improve resilience to other risks such as prevalence to pests and diseases (van Leeuwen et al., 2019).

It is important to note that other broader considerations are relevant for the selection of most appropriate local adaptive measures. The shift in sensory profiles of wines may change the concept of "terroir" and the wine typicity that some regions are well-known for (van Leeuwen and Seguin, 2006; Santos et al., 2020). Other considerations include for example socio-economic aspects, market preferences, transportation and logistics, and risks of other natural hazards that also affect the vulnerability of the NZ wine industry (Mosedale et al., 2016; Cradock-Henry and Fountain, 2019).

## 5. Conclusion

Our study has implemented two empirical modeling approaches (GFV and GSR models) to assess climate change impacts to flowering, véraison and sugar ripeness of grapevines in New Zealand, with uncertainty represented by six GCMs and four RCPs. Results indicate that warmer temperatures due to climate change are likely to advance the phenological stages of grape vines in New Zealand. Specifically, grapes will reach flowering and véraison at earlier dates which influences timing and subsequent berry ripening as demonstrated by advances in sugar ripeness. Projected changes in grapevine development indicates that cultivar shifts represent a possible option to adapt to climate change. Nevertheless, compression of key phenological stages may still occur.

Further understanding phenological characteristics of a wide range of cultivars is a key aspect that should be considered for increasing resilience of the wine industry to climate change. Future research is also needed to encompass a wider range of risk factors, identifying vulnerability, sensitivity, and adaptive capacity for a well-informed industry.

## Data Availability Statement

The raw data supporting the conclusions of this article will be made available by the authors, without undue reservation.

## Author Contributions

A-GA and AP: substantial contributions to the conception of the work and drafting the work. A-GA and RL: substantial contributions to the analysis of the work. AS provided critical climate change datasets. ET provided review, writing, and editing input. All authors critically revised the manuscript for important intellectual content. All authors agreed to be accountable for the content of the work.

## Conflict of Interest

The authors declare that the research was conducted in the absence of any commercial or financial relationships that could be construed as a potential conflict of interest. The handling editor declared a shared affiliation with one of the authors AP at the time of the review.
