# Supplementary material for: Projected Wine Grape Cultivar Shifts Due to Climate Change in New Zealand
Source: Front Plant Sci. 2021 Apr 21;12:618039. doi: 10.3389/fpls.2021.618039 (PMC8099174; doi:10.3389/fpls.2021.618039)
Supplement: Supplementary file 1 [file Data_Sheet_1.PDF]

Supplementary Material

To identify land that we considered suitable for viticulture, we combined two vector datasets, the New Zealand Land Cover Database (LCDB) (Manaaki Whenua – Landcare Research, 2004) with the land use capability (LUC) from the New Zealand Land Resource Inventory (Manaaki Whenua – Landcare Research, 2018). We excluded classes 3w, 4w, 5w, and all others with an LUC class ≥ 6 as areas not suitable for viticulture because the wetness index was too high. The target resolution of outputs is determined by the resolution of the available input climate model data. Since this resolution is relatively low ($0.05°$) and the published LUC dataset has a higher resolution reference scale (1:50 000), the method to determine a pixel's association with a dominant LUC class requires explanation.

We first rasterized the LUC vector dataset to a $0.001°$ grid using the same coordinate reference system as the climate data. This rasterization process also included a binary classification of the dataset: each output cell is either compatible (1) or incompatible (0) with viticulture, according to the class exclusion rule already mentioned. We then downsampled the binary raster dataset with a resampling algorithm that took the 75th percentile value: an output cell is therefore only considered compatible with viticulture if 25% or more of the input cells were considered compatible. This is demonstrated in Figure S1. For the GFV and GSR models, and for all statistics mentioned in this paper, analysis was only applied to pixels that met this classification.

REFERENCES

[Dataset] Manaaki Whenua – Landcare Research (2004). New Zealand Landcover Database (LCDB) (version 2). https://koordinates.com/layer/1072-land-cover-database-version-2-lcdb2

[Dataset] Manaaki Whenua – Landcare Research (2018). New Zealand Land Resource Inventory (NZLRI) Land Use Capability (revision 5). https://lris.scinfo.org.nz/layer/48076-nzlri-land-use-capability

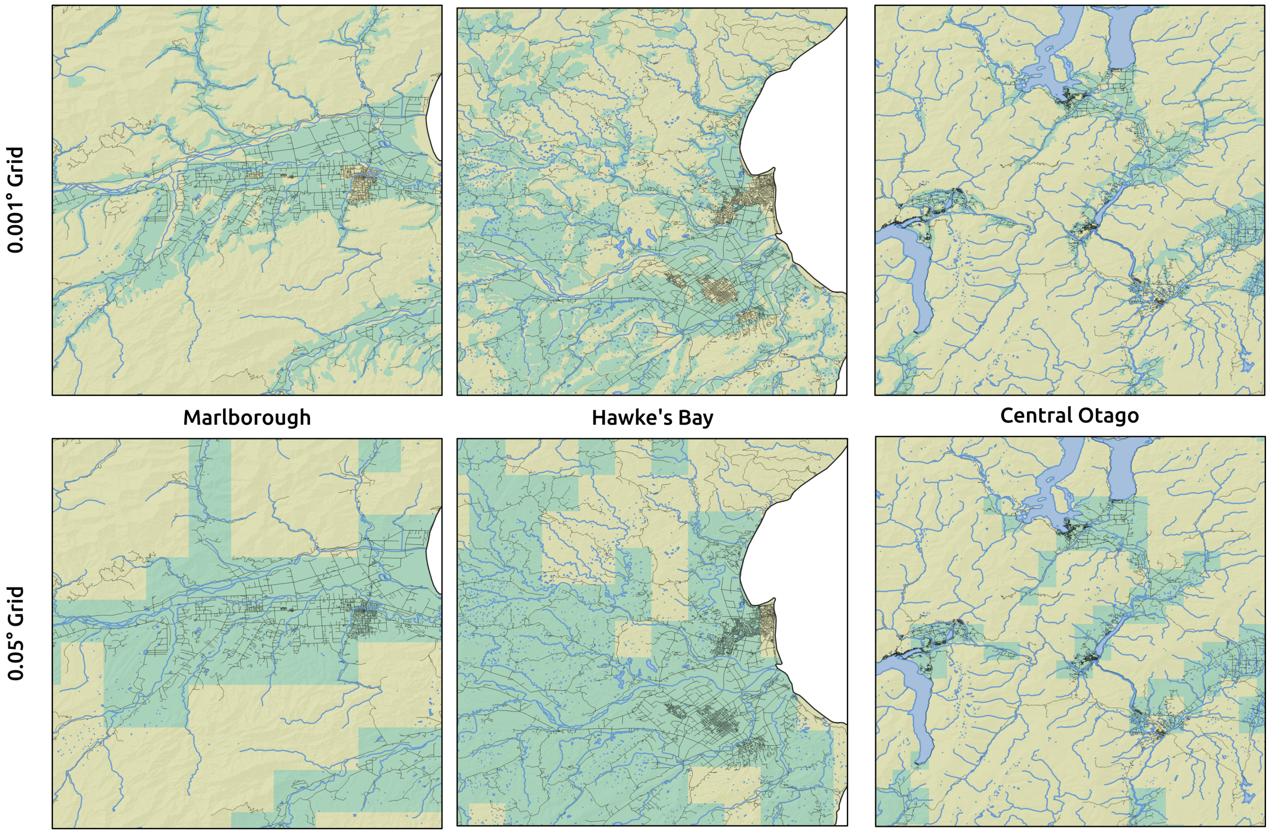

Figure S1. Major viticultural regions of New Zealand, demonstrating the difference between the 0.001° grid at which land use capability was converted into a binary classification, and the 0.05° target grid. Yellow indicates areas that are considered incompatible with viticulture, and green indicates areas that are compatible. White striped areas represent vineyards as at 2004. The final areas will be those that are both compatible, and proximate to the 2004 vineyards. Note that the three regions are not presented at equal scales.

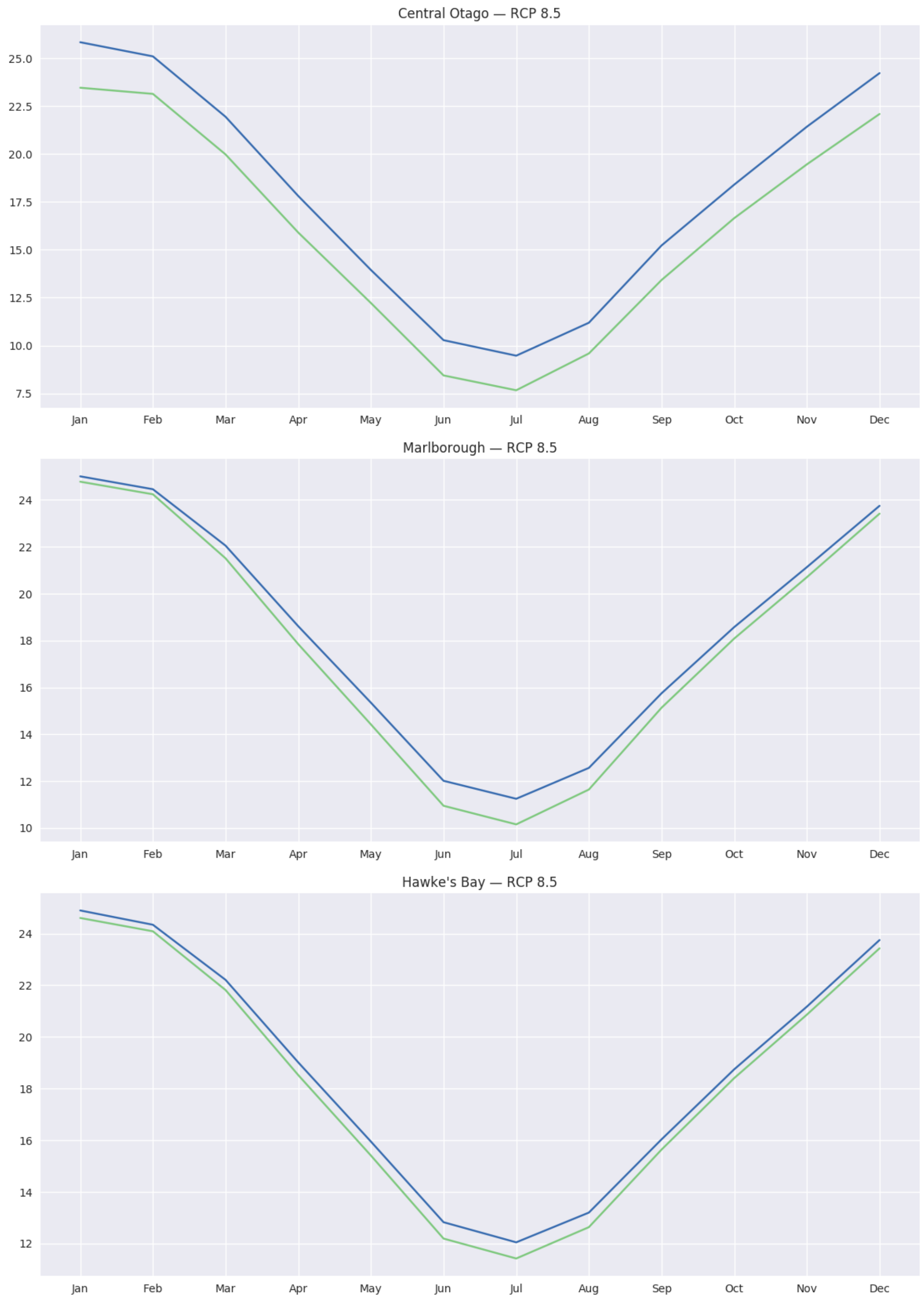

Figure S2. Mean Monthly temperature change from current (1986–2005 in green) to end of century (2081–2100 in blue) for RCP8.5.

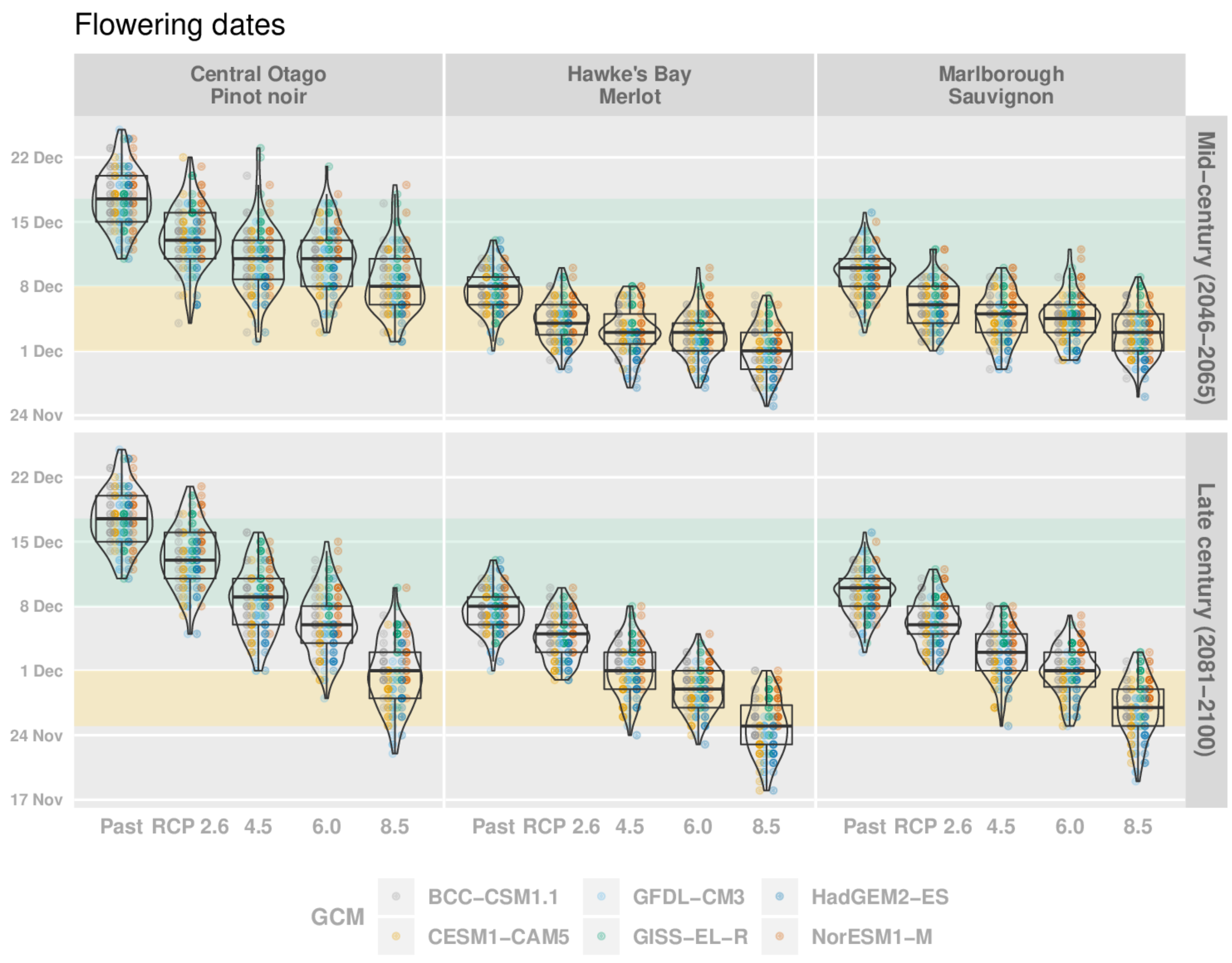

Figure S3. Predicted flowering dates for three regional cultivars: Merlot in Hawke's Bay, Pinot noir in Central Otago, and Sauvignon blanc in Marlborough for mid-century and end of the century and six GCMs.

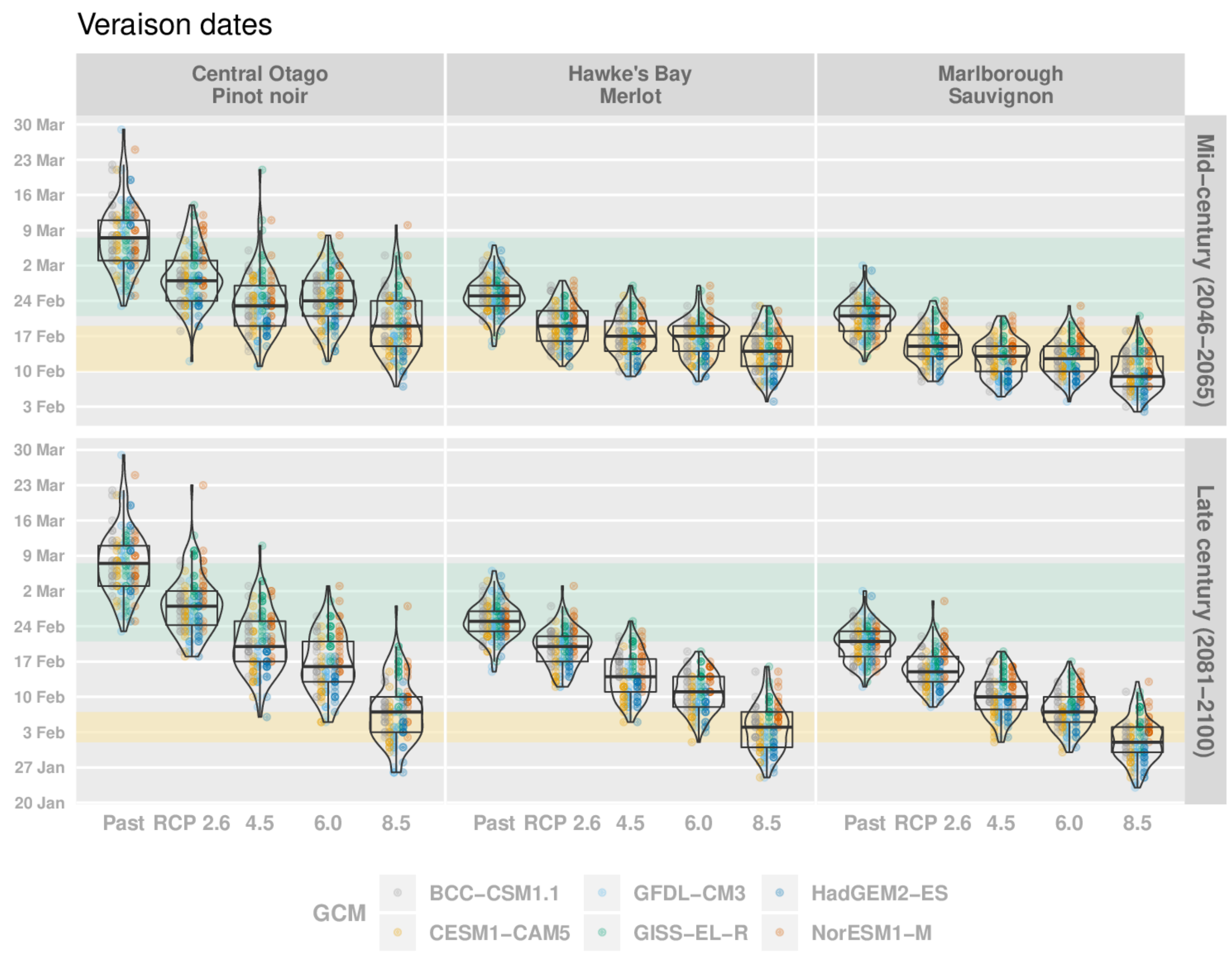

Figure S4. Predicted véraison dates for three regional cultivars: Merlot in Hawke's Bay, Pinot noir in Central Otago, and Sauvignon blanc in Marlborough for mid-century and end of the century and six GCMs.

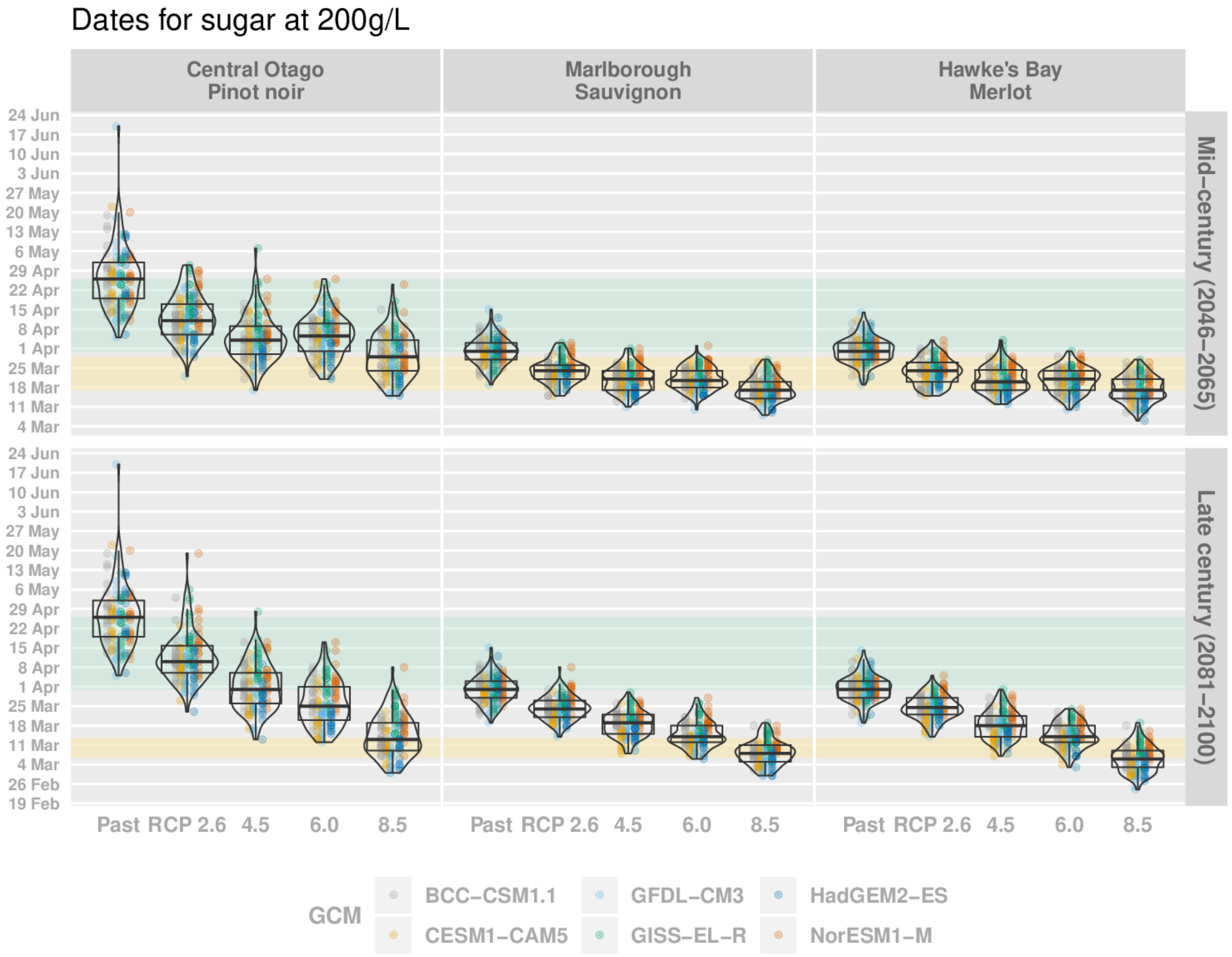

Figure S5. Predicted sugar ripeness (target sugar content reaching 200 g/l) dates for three regional cultivars: Merlot in Hawke's Bay, Pinot noir in Central Otago, and Sauvignon blanc in Marlborough for mid-century and end of the century and six GCMs.

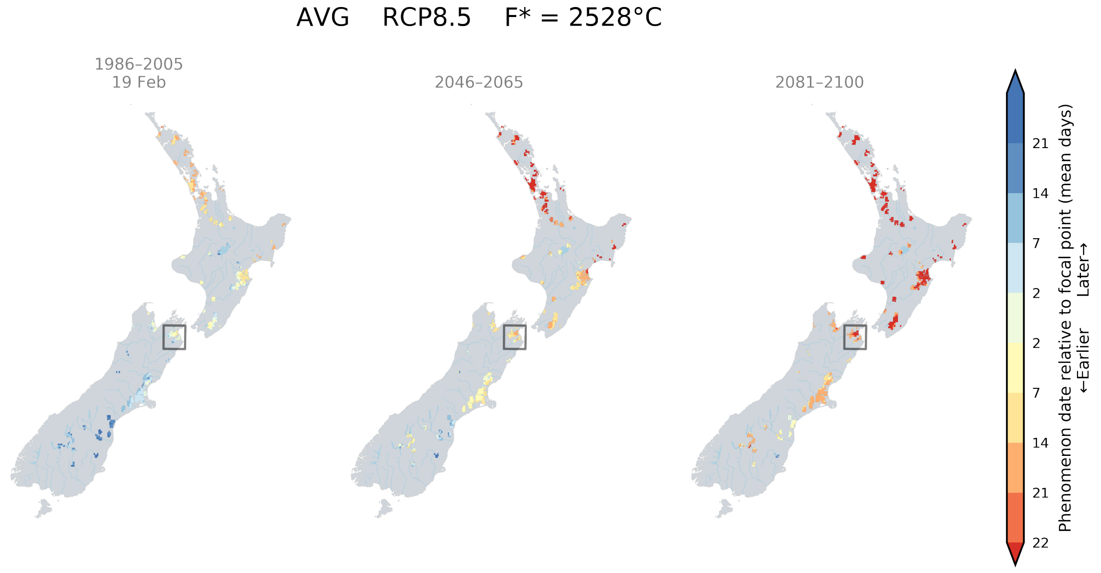

Figure S6. Relative difference in véraison date from Marlborough (average date of 19 February for Sauvignon blanc)

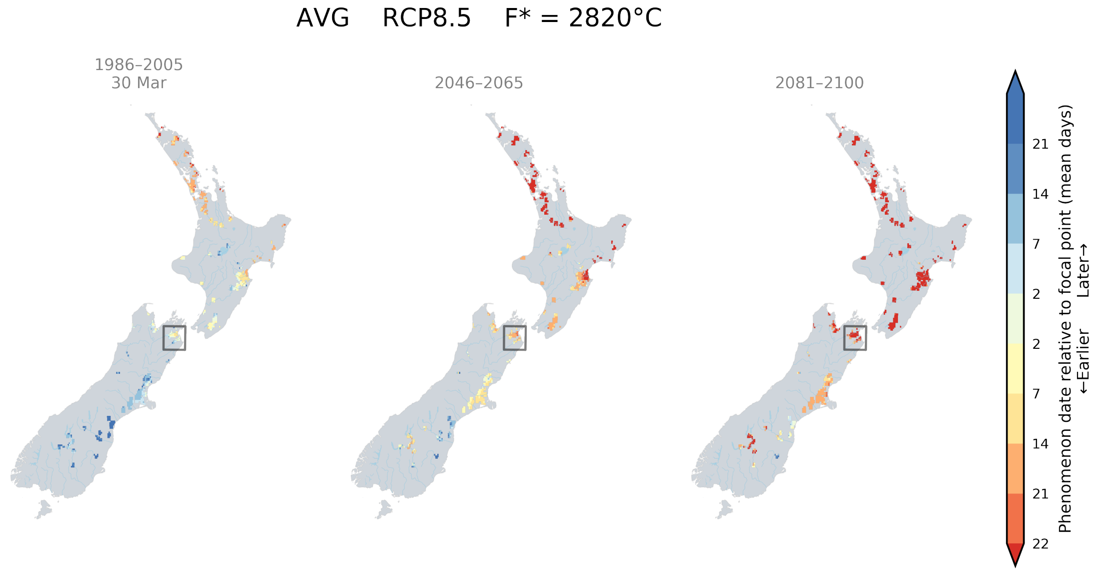

Figure S7. Relative difference in sugar target ripeness (200 g/l) date from Marlborough (average date of 30 March for Sauvignon blanc).

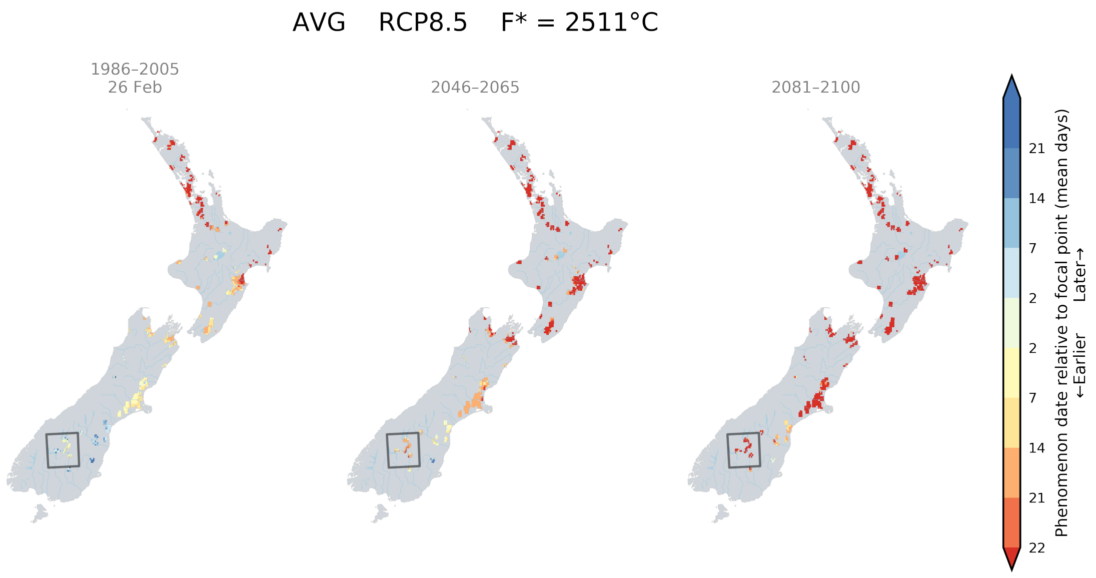

Figure S8. Relative difference in véraison date from Central Otago (average date of 26 February for Pinot noir).

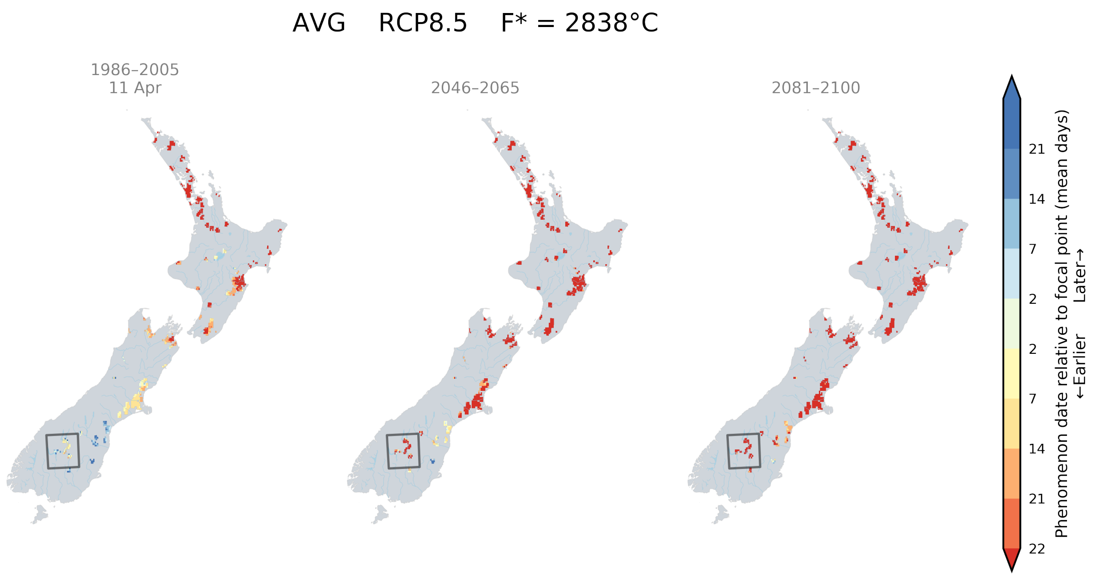

Figure S9. Relative difference in sugar target ripeness (200 g/l) date from Central Otago (average date of 11 April for Pinot noir).

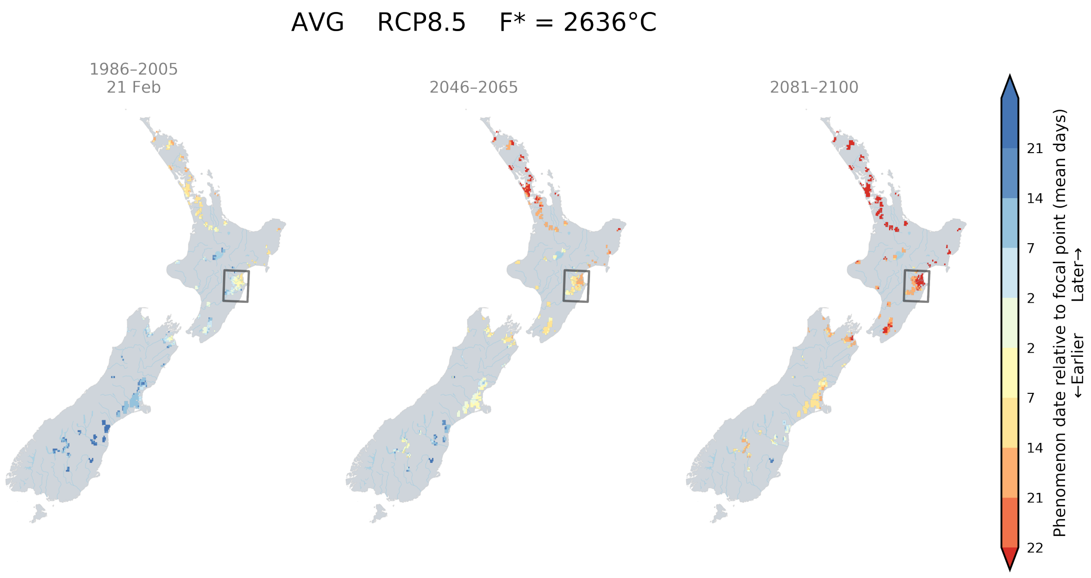

Figure S10. Relative difference in véraison date from Hawke's Bay (average date of 21 February for Merlot).

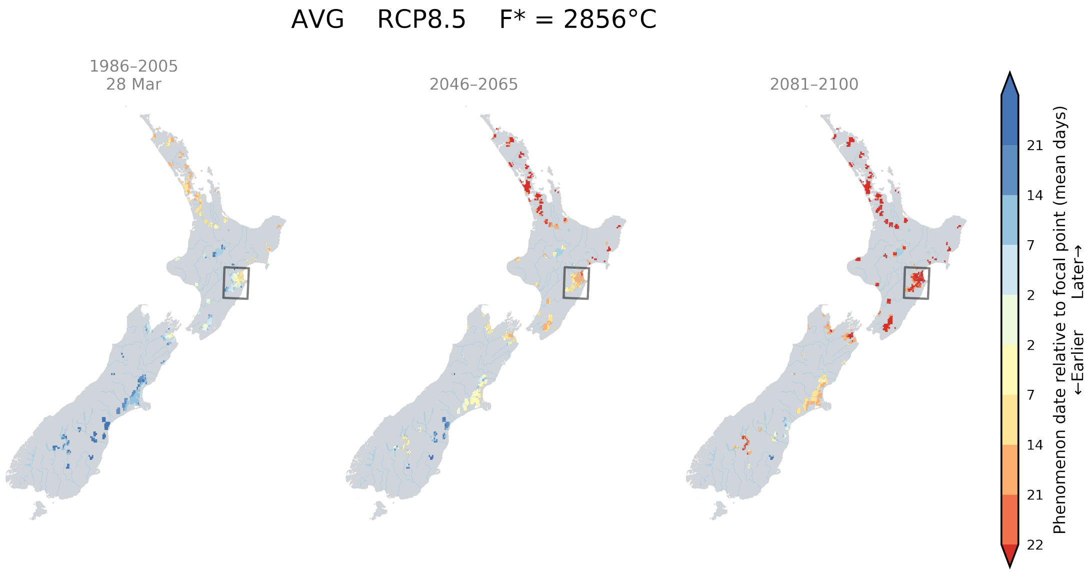

Figure S11. Relative difference in sugar target ripeness (200 g/l) date from Hawke's Bay (average date of 28 March for Merlot).
